# Contact Toxicity, Antifeedant Activity, and Oviposition Preference of Osthole against Agricultural Pests

**DOI:** 10.3390/insects14090725

**Published:** 2023-08-24

**Authors:** Fang Dong, Xin Chen, Xingyuan Men, Zhuo Li, Yujun Kong, Yiyang Yuan, Feng Ge

**Affiliations:** 1 Xinjiang Production and Construction Corps Key Laboratory of Special Fruits and Vegetables Cultivation Physiology and Germplasm Resources Utilization, College of Agriculture, Shihezi University, Shihezi 832003, China; dongfang_1012@foxmail.com; 2 Institute of Plant Protection, Shandong Academy of Agricultural Sciences, Jinan 250100, China; menxy2000@hotmail.com (X.M.); lizhuo0613@163.com (Z.L.); 3 College of Life Sciences, Cangzhou Normal University, Cangzhou 061001, China; 13682053007@163.com; 4 School of Life Sciences, Institute of Life Sciences and Green Development, Hebei University, Baoding 071002, China; kongyujun0701@foxmail.com

**Keywords:** botanical pesticide, osthole, antifeedant, oviposition preference, *Tetranychus urticae*, *Myzus persicae*, *Bactrocera dorsalis*

## Abstract

**Simple Summary:**

The excessive utilization of chemical pesticides has resulted in the emergence of pesticide resistance, the disturbance of ecosystems, environmental degradation, and health hazards. Botanical pesticides offer a promising alternative to chemical pesticides, as they have minimal impacts on human health and the environment. Osthole, which has shown pesticidal activities, is the primary bioactive compound in *Cnidium monnieri* (L.) Cusson. In this study, we assessed osthole for its contact toxicity, antifeedant activity, and oviposition preference against three agricultural pests: the *Tetranychus urticae*, *Myzus persicae*, and *Bactrocera dorsalis*, using laboratory bioassays. Osthole exhibited pesticidal effects against the *T. urticae* and *M. persicae*, impeding their fecundity and development. Additionally, it demonstrated significant antifeedant activity against the *T. urticae* and acted as a potent attractant for oviposition by the *B. dorsalis*. These findings highlight and expand the potential of osthole as a botanical pesticide for the control of these agricultural pests.

**Abstract:**

Osthole, the dominant bioactive constituent in the *Cnidium monnieri*, has shown acute pesticidal activities. However, its detailed toxicity, antifeedant, and oviposition preference effects against agricultural pests have not been fully understood, limiting its practical use. This study aimed to investigate the contact toxicity, antifeedant activity, and oviposition preference of osthole against three agricultural pests (*Tetranychus urticae*, *Myzus persicae*, and *Bactrocera dorsalis*). Our results showed that the *Cnidium monnieri* (L.) Cusson (CMC) has a high osthole content of 11.4 mg/g. Osthole exhibited a higher level of acute toxicity against the *T. urticae* to four other coumarins found in CMC. It showed significant pesticidal activity against *T. urticae* and *M. persicae* first-instar nymphs and adults in a dose-dependent manner but not against *B. dorsalis* adults. Osthole exposure reduced the fecundity and prolonged the developmental time of the *T. urticae* and *M. persicae*. Leaf choice bioassays revealed potent antifeedant activity in the *T. urticae* and *M. persicae*. Furthermore, the female *B. dorsalis* showed a distinct preference for laying eggs in mango juice with 0.02 mg/mL osthole at 48 h, a preference that persisted at 96 h. These results provide valuable insights into the toxicity, repellent activity, and attractant activity of osthole, thereby providing valuable insights into its potential efficacy in pest control.

## 1. Introduction

The intensive use of chemical pesticides has led to the development of pesticide resistance, ecosystem imbalances, environmental deterioration, and health risks [1,2]. The botanical pesticides, with reduced health and environmental impacts, are promising alternatives to chemical pesticides [3,4].

*Cnidium monnieri* (L.) Cusson (CMC) is the dried fruit of the *C. monnieri* plant, which is primarily cultivated in China, Japan, Korea, and Vietnam (source: https://www.gbif.org/species/3034720, accessed on 15 June 2023). It is commonly recognized and utilized in traditional Chinese medicine due to its extensive array of pharmaceutical properties [5]. CMC contains 429 chemical constituents, including coumarins, volatile constituents, liposoluble compounds, chromones, monoterpenoid glucosides, terpenoids, glycosides, and glucides [6]. Coumarins are major chemical compounds in CMC, among which osthole is the dominant bioactive constituent with antitumor, anti-inflammatory, neuroprotective, osteogenic, cardiovascular protective, antimicrobial, and antiparasitic activities [7,8,9,10].

Osthole (7-methoxy-8-(3-methyl-2-butenyl) coumarin), a simple coumarin, is mainly found in the genera of Umbelliferae and Rutaceae [11,12]. Recent research has highlighted its potential as an botanical insecticide, as it displays efficacy against green peach aphids (*Myzus persicae*) and two-spotted spider mites (*Tetranychus urticae*) [13]. Osthole degrades rapidly in plant leaves and is considered safe for natural predators [13]. However, its detailed toxicity, antifeedant, and oviposition preference effects against agricultural pests remain incompletely understood, significantly limiting its practical application.

The *T. urticae*, *M. persicae*, and oriental fruit fly (*Bactrocera dorsalis*) are important agricultural pests that feed on hundred kinds of fruits and vegetables [14,15,16]. They can destroy many economically valuable host plants in agriculture and horticulture, including tomatoes, peppers, cucumbers, strawberries, maize, soy, apples, bananas, mango, grapes, and citrus fruits [17,18,19]. The excessive reliance on chemical pesticides for controlling these pests not only contributes to the development of high resistance but also poses significant risks to the environment and human health [20,21,22,23,24,25]. Coumarins, a class of natural compounds, have been widely recognized for their strong pesticidal properties. One notable example is scoparone, which was originally isolated from the Chinese herbal medicine *Artemisia capillaris*. Scoparone is a phenolic coumarin known for its potent acaricidal activity, achieved through its ability to modulate downstream Ca^2+^ signaling pathways [26]. 

This study aims to evaluate the efficacy of osthole against the *T. urticae*, *M. persicae*, and *B. dorsalis*. The assessment will specifically focus on its contact toxicity, antifeedant activity, and oviposition preference. The results obtained will shed light on the potential agricultural applications of osthole.

## 2. Materials and Methods

### 2.1. Arthropods and Reagents

A colony of *T. urticae* was collected from a strawberry field population during November 2021 in Shandong Province, China, and subsequently raised on caged common-bean (*Phaseolus vulgaris*). The *M. persicae* (green clone) used in this study were derived from a wild population collected in the oilseed rape (*Brassica napus*) fields of Shandong province, China, during March 2022. Aphids were reared under laboratory conditions on caged Chinese cabbage (*B. campestris*). *B. dorsalis*, which were originally obtained from a continuously maintained culture at the Institute of Plant Protection, Shandong Academy of Agricultural Sciences, were maintained by rearing larvae on bananas and maintaining adults in cages with an artificial diet consisting of yeast extract and sugar. All arthropods were maintained under controlled conditions at 25 ± 1 °C with a photoperiod of 16:8 h (L:D) until use in the experiments.

CMC was purchased from Binnong Technology Co., Ltd. (Wudi, China), while osthole with a purity of 99.5% and Dimethylsulfoxide (DMSO) and Triton X-100 were, respectively, obtained from the National Institutes for Food and Drug Control, Sinopharm, Chemical Reagent Co., Ltd. (Shanghai, China), and Solarbio Co., Ltd. (Beijing, China). The reference compounds used in this study included imperatorin (98%), methoxsalen (99.98%), xanthotoxol (98%), columbianadin (98%), isopimpinellin (98%), 5-methoxypsoralen (98%), meranzin (hydrate) (98%), angelicin (98%), auraptenol (98%), isogosferol (97%), and scoparone (98%), which were purchased from MedChemExpress (Shanghai, China), Aladdin (Shanghai, China), Yuanye (Shanghai, China), Acmec (Shanghai, China), Glpbio (Montclair, NJ, USA), Bidepharm (Shanghai, China), Macklin (Shanghai, China), and Desite (Chengdu, China), respectively.

### 2.2. Extraction and Quantification of Coumarins in CMC

Coumarins were extracted from CMC using a modified method based on Bourgaud et al. (1994) [27]. In brief, 10 g of powdered CMC was placed in sterile medical gauze and extracted three times with 100 mL of 95% ethanol using a Soxhlet extractor for 3.5 h each time. The resulting extracts were combined, filtered, and concentrated under vacuum at 50 °C. The concentrate was then dissolved in 25 mL of methanol and subjected to centrifugation at 10,000 rpm at 4 °C for 5 min. A 250 μL aliquot of the supernatant was transferred to a new tube and made up to 25 mL with methanol. Before chromatographic analysis, the solution was filtered through a 0.22 μm PTFE membrane.

The quantification of auraptenol, columbianadin, xanthotoxol, and isopimpinellin was performed using a Waters ACQUITY UPLC I-Class liquid chromatography (LC) system coupled to a TQ-S triple quadrupole mass spectrometer (MS) with an electrospray ionization (ESI) source (Waters Corp., Milford, MA, USA). Chromatographic separation was carried out on a Waters AcQuity UPLC BEH C18 column (4.6 mm × 150 mm, 1.7 μm) with an injection volume of 2 μL for all samples and a mobile phase flow rate of 5 μL/min. The gradient elution was performed using 0.1% fomic acid (A) and acetonitrile (B) with the following conditions: 0–0.5 min, 30% B; 0.5–1 min, 30% B to 90% B; 1–4 min, 90% B; and 4–5 min, 90% B—30% B. Quantification was conducted in the positive ion multiple reaction monitoring (MRM) mode with a capillary voltage of 3.0 kV, desolvation temperature of 500 °C, and flow rates of 850 and 150 L/h for desolvation and cone gas, respectively.

Osthole, imperatorin, isopimpinellin, 5-methoxypsoralen, methoxsalen, meranzin (hydrate), angelicin, and isogosferol were quantified using a Shimadzu Nexera X2 LC-30AD liquid chromatography (LC) system coupled to an AB SCIEX TRIPLE QUAD 4500 mass spectrometer (MS) with an electrospray ionization (ESI) source (Shimadzu Corp., Chiyoda-ku, Tokyo, Japan). Chromatographic separation was carried out on an Angilent Poroshell 120 EC C18 column (4.6 mm × 100 mm, 2.7 μm) with an injection volume of 5 μL and a mobile phase flow rate of 0.4 mL/min. A gradient elution consisting of 0.1% formic acid (A) and Methanol (B) was used to separate the coumarins, with the following gradient conditions: 0–0.2 min, 10% B; 0.2–2 min, 10% B to 90% B; 2–8 min, 90% B; and 8–8.1 min, 90% B—10% B. Quantification was performed in the positive ion multiple reaction monitoring (MRM) mode with two MRM transitions, Q1 (precursor ion) and Q3 (product ion), and a dwell time of 50 ms for the quantitation and identification of the coumarins based on mass-to-charge ratio (*m*/*z*) calculations.

### 2.3. Acute Toxicity Bioassay

The acute toxicity bioassays of all coumarins against the *T. urticae* or *M. persicae* were conducted using a previously described leaf-dipping method with some modifications [28]. Each coumarin was first dissolved in DMSO and then diluted with distilled water containing 0.1% Triton X-100 to varying concentrations, with the final DMSO concentration being 3% in the final dilutions. Individual 60 mm cut leaf discs, each with 20 one-day-old adult females, were dipped in dilutions for a duration of either 5 s (for *T. urticae*) or 10 s (for *M. persicae*). The excess solution was then blotted off with filter papers, and the arthropods were subsequently transferred to an agar-containing Petri dish with a new leaf disc. Arthropods treated with distilled water containing 0.1% Triton X-100 and 3% DMSO via the same method served as the negative control. Mortality was recorded every 24 h, and each treatment was repeated five times. Scoparone, specifically used as a positive control treatment, was employed in a 24 h mortality bioassay to assess the efficacy of osthole against the *T. urticae.* The larvicidal bioassays were conducted using a similar methodology, except leaf discs infested with 1st instar nymphs were immersed in osthole solutions for a duration of 10 s. Mortality was assessed 3 days post-experiment, and each treatment was repeated five times. The pesticidal activities of osthole against the *B. dorsalis* were evaluated using a modified feeding method [29]. Batches of 20 three-day-old adult flies, consisting of 10 females and 10 males, were subjected to a 6 h starvation period followed by feeding with artificial diets containing osthole. Mortality was assessed every 24 h over 7 days. Each treatment was repeated three times.

The LC_20_, LC_50_, and LC_90_ values were calculated using Probit analysis in SPSS 14.0 software. The LC_20_ and LC_50_ concentrations were subsequently used in experiments to evaluate the effects of osthole on larval growth and adult reproduction.

### 2.4. Larval Growth and Development Bioassays

To assess the effects of osthole on larval growth and development, newly hatched mite or aphid larvae were soaked in osthole dilutions (LC_20_ and LC_50_) using the previously mentioned method. The control group utilized distilled water with 0.1% Triton X-100 and 3% DMSO. In the case of the *T. urticae*, the control group consisted of 60 larvae, while the LC_20_ and LC_50_ treatment groups had 75 and 90 larvae, respectively. For the *M. persicae*, the control group used 50 larvae, with the LC_20_ and LC_50_ treatment groups having 60 and 75 larvae, respectively. Each larva was regarded as a single replication, and deceased larvae were not included in the final statistical analysis. Following treatment, the larvae were individually placed on agar-filled Petri dishes with fresh leaf discs. The duration of different developmental stages was recorded, and body lengths were measured one day after emerging.

### 2.5. Fecundity Bioassays

Newly emerged *T. urticae* and *M. persicae* gravid female adults were subjected to the osthole dilutions (LC_20_ and LC_50_) using the aforementioned leaf-dipping method. After 24 h, the surviving females were individually transferred to agar-containing Petri dishes with a fresh leaf disc. The *T. urticae* eggs and the *M. persicae* nymphs were counted and removed daily for 3 days, with ten replicates carried out for each assay.

Meanwhile, the fecundity assay for the *B. dorsalis* was conducted using a feeding method with some modifications [30]. Specifically, ten-day-old female and male flies (10 each) were placed in a plastic cup (250 mL) and fed with artificial food containing osthole at different concentrations (0.0625–2 mg/mL). The control treatment involved artificial feed without osthole. The eggs were collected with a plastic tube (10 mL) along with mango juice, and the number of eggs was counted daily for 15 days. Each treatment was repeated ten times.

### 2.6. Antifeedant Bioassays

To evaluate the antifeedant activities of osthole on the *T. urticae*, we applied osthole to one half of a 35 mm diameter common bean leaf disc using a sterile cotton swab, while the other half was treated with distilled water containing 0.1% triton X-100 and 3% DMSO as a control. After the solution dried, the leaf was transferred to a Petri dish and 30 female adult mites were introduced onto the central line, allowing mites to choose between treated and untreated control areas. The number of mites on each half of the leaf area was recorded at 0.5, 1, 2, 6, 12, 24, and 48 h intervals. Each concentration was tested with 30 mites, and eight replicates were performed.

To assess the antifeedant properties of osthole against the *M. persicae*, Chinese cabbage leaves were briefly immersed in an osthole solution for 10 s, while similarly sized leaves were submerged in distilled water containing 0.1% triton X-100 and 3% DMSO as a control. The treated and control leaves were transferred to plastic boxes (10 × 16 cm) containing a 1 cm thick hydrophilic sponge, 1 mm thick filter paper, and black PVC. Cohorts of one-day-old adult female aphids were introduced onto the central axis that bisected the experimental arena into two equal halves. The number of aphids on each leaf was recorded at 0.5, 1, 2, 6, 12, 24, and 48 h intervals. Each concentration was tested on 30 aphids with eight replicates.

### 2.7. Oviposition Preference Bioassays

The oviposition preference of the *B. dorsalis* was assessed using a modified version of the methodology described by Li et al. (2020) [31]. Egg collection devices were created by perforating 180 mL plastic cups with ten rows of ten holes each, positioned 5 cm from the bottom of the cup. The diameter of each hole was approximately 1 mm, with a spacing of about 0.5 cm between them. Each row was spaced at around 1.3 cm intervals. Two devices were placed in a sterilized cage (60 × 35 × 35 cm) containing 15 female and 15 male flies to inoculate eggs. One device contained a solution of osthole in mango juice with 3% DMSO, while the other served as a control and contained only a mango juice solution with 3% DMSO. The surface of each device was also sprayed with the corresponding mango juice solutions. The number of eggs was recorded at 24, 48, and 72 h intervals. The experiment was repeated eight times for each treatment.

### 2.8. Data Analysis

We used ANOVA and Tukey’s HSD multiple comparison tests to determine differences in coumarin content in CMC. The simple linear regression analysis was performed to designate the mortality rates of *T. urticae* and *M. persicae* nymphs against osthole concentrations. ANOVA and Tukey’s HSD tests were also used to identify differences in the osthole’s impact on developmental period, body length, and reproduction. The Chi-squared test was employed to analyze the antifeedant activity of osthole on female *T. urticae* and *M. persicae* adults, as well as the differences in the oviposition attraction of osthole on female *B. dorsalis* adults. All analyses were performed in GraphPad Prism 9.

## 3. Results

### 3.1. Osthole Is the Primary Coumarin in CMC

**Figure 1:**
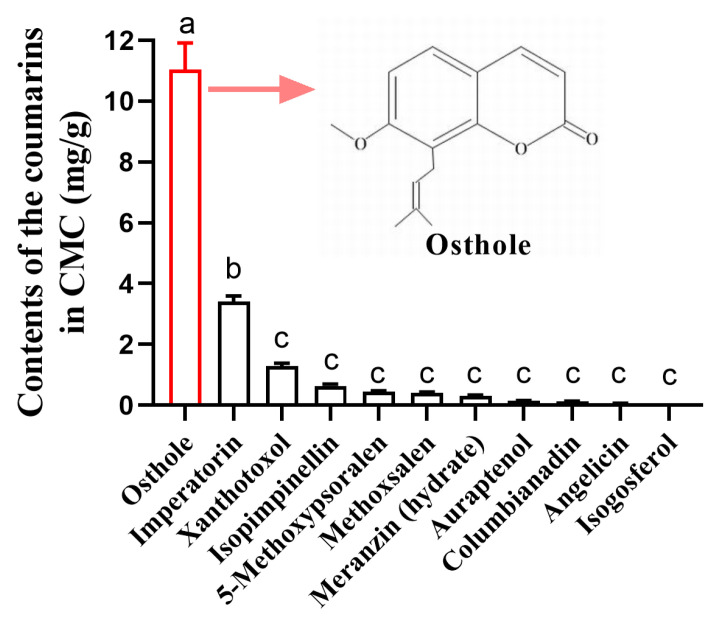
Contents of the coumarins in CMC. Different lowercase letters indicate significant differences among coumarins (one-way analysis of variance (ANOVA), followed by multiple comparisons of Tukey’s test, *p* < 0.05). Error bars represent standard error (*n* = 4).

Based on LC/MS analysis of CMC, osthole was found to be the most abundant coumarin in CMC, accounting for 67.1% of the total 11 coumarins determined, followed by imperatorin and xanthotoxol (Figure 1, Table S1). The contents of isopimpinellin, 5-methoxypsoralen, methoxsalen, meranzin (hydrate), auraptenol, and columbianadin ranged from 0.12 to 3.41 mg/g (Figure 1). CMC only contains trace amounts of angelicin and isogosferol (Figure 1).

### 3.2. Osthole Showed Comparable Contact Toxicity to Other Coumarins in CMC against T. urticae

**Figure 2:**
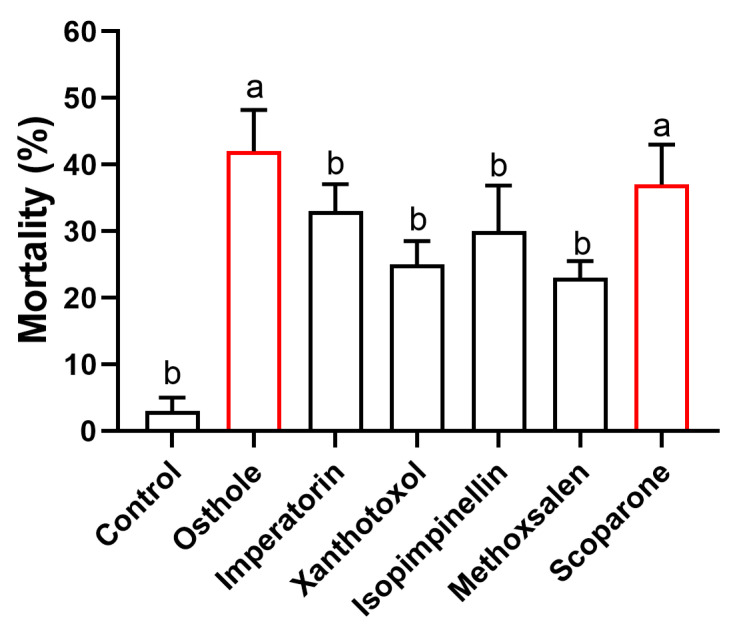
Comparison of 24 h mortality in adult *T. urticae* exposed to various 2 mg/mL coumarins. Different lowercase letters indicate significant differences among coumarins (Kruskal–Wallis test), followed by multiple comparisons of Dunn’s test, *p* < 0.05). Error bars represent standard error (*n* = 20).

The mortality rates of the adult *T. urticae* varied between 23% and 42% across all six coumarins investigated (Figure 2). It is noteworthy that both osthole and scoparone exhibited significantly higher mortality rates compared to the remaining coumarins. Specifically, osthole treatment resulted in a mortality rate of 42%, while scoparone treatment led to a mortality rate of 37%.

### 3.3. Osthole Exhibits Toxicity towards T. urticae and M. persicae but Not B. dorsalis

**Figure 3:**
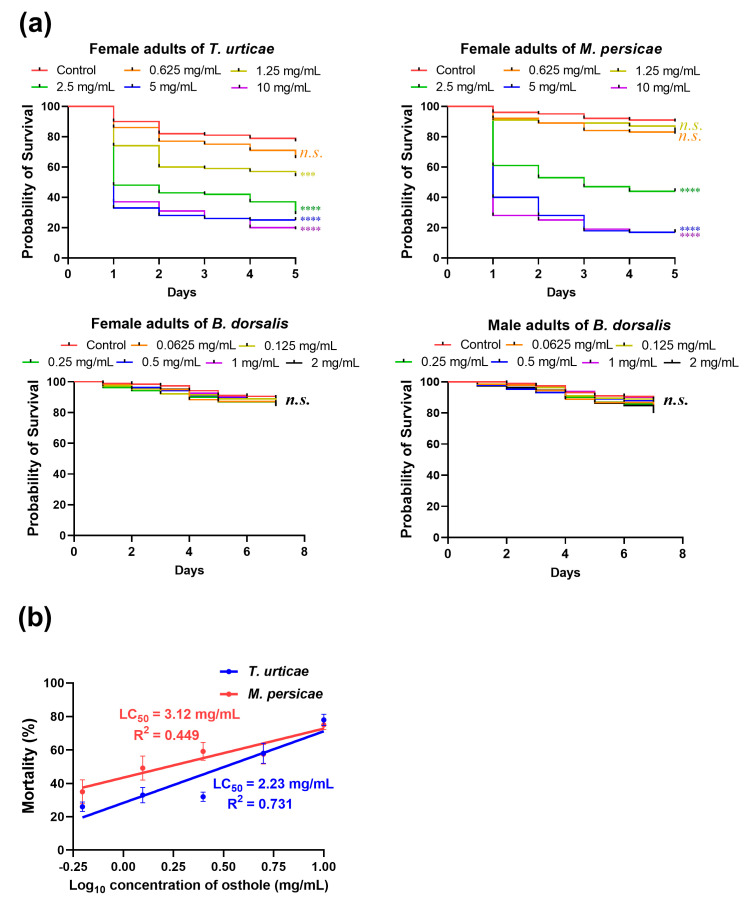
The acute toxicity of osthole against *T. urticae*, *M. persicae*, and *B. dorsalis*. (**a**) Kaplan–Meier survival curve of adults from the three pest species upon exposure to osthole (*n* = 20). The asterisk indicates a statistically significant difference measured using Log-Rank test as compared to the wild type (***, *p* < 0.0005; ****, *p* < 0.0001; *n.s.*, not significant). (**b**) Concentration–mortality lines for the mortality of *T. urticae* and *M. persicae* nymphs after osthole exposure (*n* = 5 for *T. urticae*, *n* = 6 for *M. persicae*). Error bars represent standard error.

The Kaplan–Meier 5-day survival curves depicted a significant decrease in the survival rates of the *T. urticae* and *M. persicae* when exposed to concentrations exceeding 1.25 and 2.5 mg/mL of osthole, respectively (Figure 3a). Specifically, the lowest survival rates were observed on day 5 at concentrations of 10 mg/mL for the *T. urticae* and 5 mg/mL for the *M. persicae*. The LC_20_ and LC_50_ values of the *T. urticae* and *M. persicae* were both dependent on the time after immersion, indicating the delayed mortality effect of osthole, as shown in Table 1 and Table 2.

The results of the simple linear regression analysis revealed significant toxicity of osthole against both the *T. urticae* (F = 62.6, df = 1, 23, *p* < 0.0001) and *M. persicae* (F = 22.83, df = 1, 28, *p* < 0.0001) (Figure 3b). The LC_20_ and LC_50_ values for *T. urticae* nymphs were 29% and 8% lower, respectively, than those of adult mites, as shown in Table 3. Similarly, the LC_20_ and LC_50_ values for *M. persicae* nymphs decreased by 85% and 29%, respectively, compared to adults (Table 3). These findings suggest that the *T. urticae* is more susceptible to osthole than the *M. persicae*, whereas adults in both arthropod species exhibit greater tolerance to osthole than nymphs.

No significant difference in survival rates was observed between the experimental and control groups after a 7-day period of feeding *B. dorsalis* adults artificial diets with varying concentrations of osthole (Figure 3a). It is worth noting that female *B. dorsalis* adults deposit their eggs within the flesh of soft fruits, and the larvae subsequently hatch and feed inside the fruits [32]. Therefore, it was important to evaluate the toxicity of osthole to *B. dorsalis* adults, but the results suggest that it may not be effective for controlling this pest.

### 3.4. The Development and Reproduction of T. urticae and M. persicae Were Impaired upon Osthole Exposure

**Figure 4:**
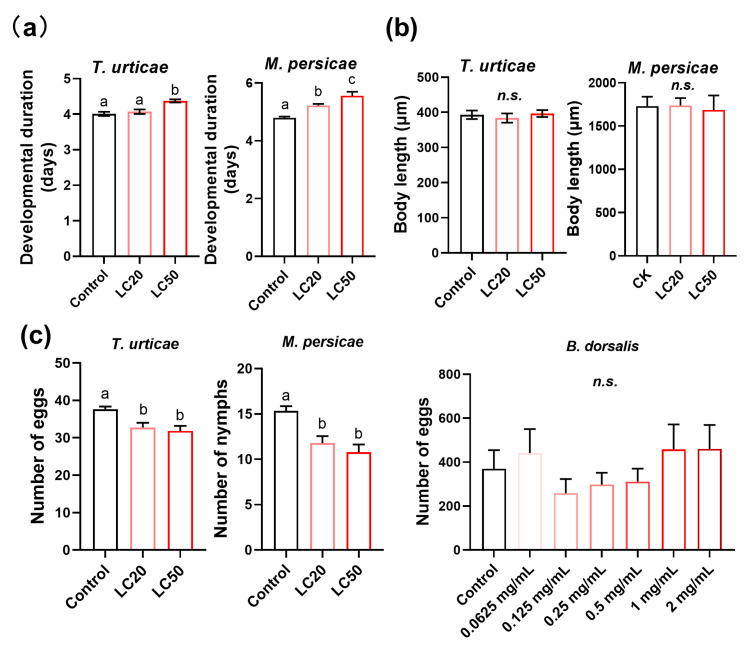
Effects of osthole treatments on the developmental period (**a**), body length (**b**), and reproduction (**c**) of *T. urticae*, *M. persicae*, and *B. dorsalis*. Different lowercase letters indicate significant differences among treatments (one-way analysis of variance (ANOVA), followed by multiple comparisons of Tukey’s test, *p* < 0.05). *n.s.* indicates not significant. Error bars represent standard error.

We found that exposure to the LC_50_ concentration of osthole significantly prolonged the larval developmental period of the *T. urticae* by 8.32%. Meanwhile, the developmental time of the *M. persicae* was significantly increased by 8.92% and 15.9% after treating them with the LC_20_ and LC_50_ concentrations, respectively, when compared to the control group (Figure 4a). However, the body lengths of both arthropods remained unchanged (Figure 4b). The fecundity of adults also significantly decreased after treatment with the LC_20_ and LC_50_ concentrations of osthole, by around 15% for the *T. urticae* and 28% for the *M. persicae* (Figure 4c). Exposure to osthole did not cause any adverse effects on the *B. dorsalis*, as indicated by the results in Figure 4c. These results indicate that osthole significantly impairs the development and reproduction of both the *T. urticae* and *M. persicae*.

### 3.5. Osthole Present Antifeedant Activity against T. urticae

**Figure 5:**
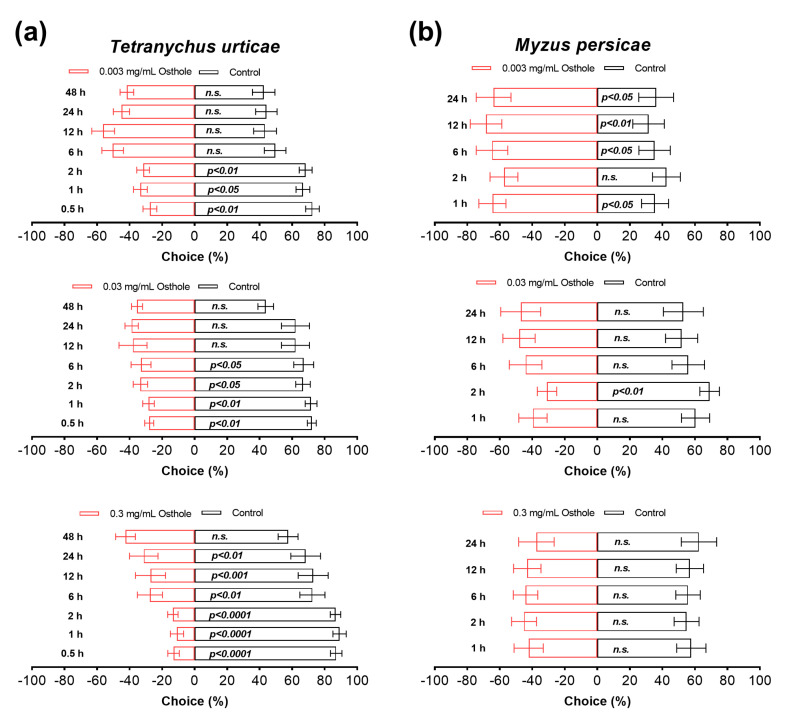
Antifeedant activity of osthole on *T. urticae* (**a**) and *M. persicae* (**b**) female adults. The Chi-squared test was used for statistical analysis. All experiments were reproduced 8 times. *n.s.* indicates not significant.

Osthole exhibited potent and concentration-dependent antifeedant activities against the *T. urticae* (Figure 5). At 0.5 h after the release of the *T. urticae*, the number of mites occupying leaves immersed in 0.003 mg/mL osthole was significantly lower compared to control leaves, with this antifeedant effect persisting for a total of 2 h (~70% versus ~30%) (Figure 5a). Increasing the concentration of osthole to 0.03 mg/mL extended the antifeedant effect for up to 6 h (Figure 5a). Similarly, a higher concentration of osthole at 0.3 mg/mL resulted in an even more pronounced and longer response, with the effect lasting for up to 24 h (Figure 5a). Notably, in the latter case, the antifeedant effects were even stronger, as a majority of the mites chose control leaves over treated ones (~80% versus ~20%) (Figure 5a). However, the antifeedant activity of osthole was observed only for the *M. persicae* at a concentration of 0.003 mg/mL, and this activity persisted for a duration of 24 h (Figure 5b).

### 3.6. Osthole Elicited Oviposition Attraction of B. dorsalis

**Figure 6:**
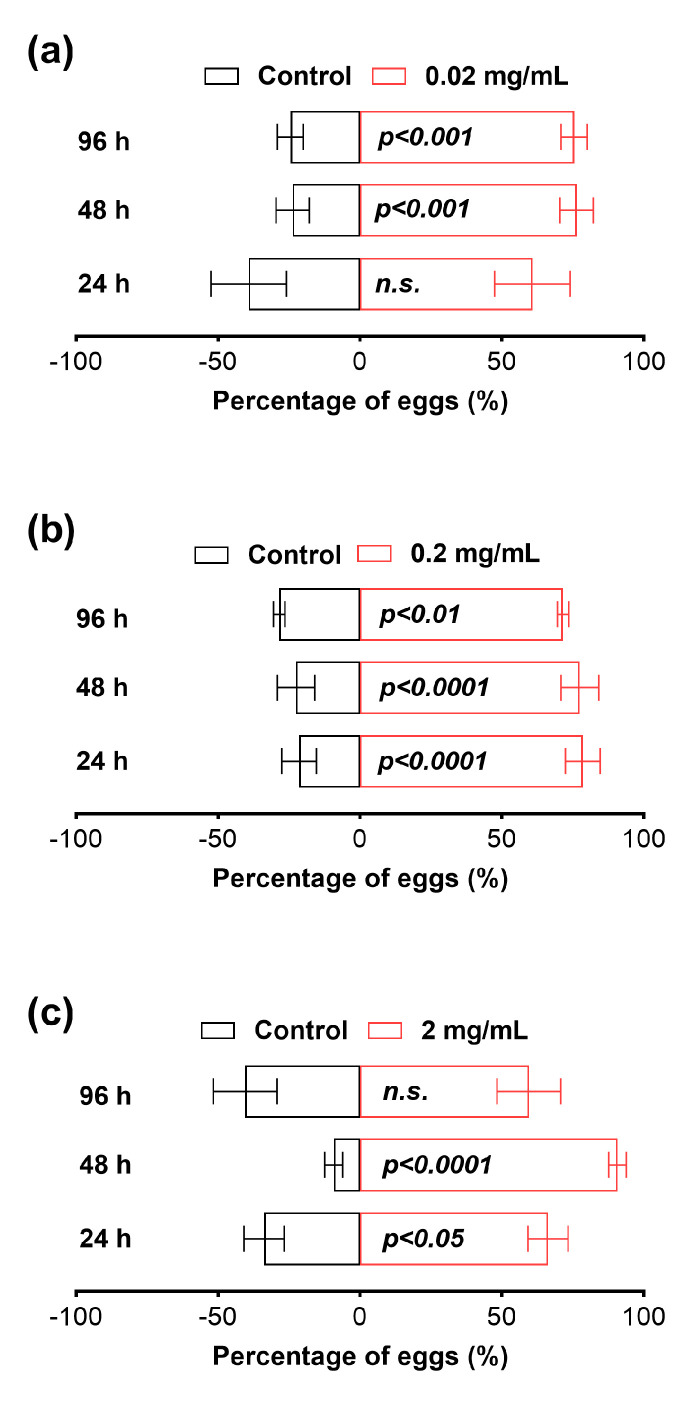
Oviposition attraction of 0.02 (**a**), 0.2 (**b**), and 2 (**c**) mg/mL osthole to *B. dorsalis*. The Chi-squared test was used for statistical analysis. All experiments were replicated five times. *n.s.* indicates not significant.

Our results revealed that female flies exhibited a preference for laying their eggs in mango juice infused with osthole at a concentration of 0.02 mg/mL after 48 h, and this preference remained evident even at the 96 h mark (Figure 6). Interestingly, when we increased the osthole concentration to 0.2 mg/mL this preference was observed as early as the 24th hour (Figure 6). Furthermore, when the osthole concentration was further increased to 2 mg/mL the preference emerged at the 24th hour and persisted until the 48th hour but subsequently diminished by the 96th hour (Figure 6).

## 4. Discussion

Previous researches have reported that CMC contains 67 types of coumarins, though the majority of their individual contents remain unknown [6]. In our study, we quantified 11 types of coumarins, including three simple coumarins, six linear furanocoumarins, and two angular furanocoumarins. Osthole was found to be the most abundant type of coumarin present (11.04 mg/g), which is consistent with previous findings [33,34]. Imperatorin content in CMC is reported to range from 2 to 9 mg/g, while the contents of isopimpinellin and 5-methoxypsoralen range from 1 to 4 mg/g [33,34,35]. In our study, we found the contents of imperatorin, isopimpinellin, and 5-methoxypsoralen in CMC from Wudi (Shandong province) to be 3.41, 0.64, and 0.45 mg/g, respectively. *C. monnieri* is widely cultivated in various regions of China, but whether the contents of coumarins in CMC exhibit quantitative variation depending on the geographic region still need further study. Furthermore, our study is the first to report the contents of the remaining seven coumarins in CMC. In CMC, coumarins are considered the primary bioactive compounds based on previous research studies [7,8,9,10]. Our study focused on evaluating the toxicity of osthole and four other coumarins that can be found in CMC against the *T. urticae*. Additionally, we included scoparone in our analysis, as it is a well-known coumarin with strong acaricidal properties against mites [26]. Interestingly, we observed that both osthole and scoparone exhibited higher toxicity against the mites compared to the other coumarins tested. This finding provides further evidence of the potential pesticidal role of osthole in CMC. Further research is necessary to assess the toxicity of combinations of coumarins against pests and evaluate their potential as effective pesticides.

Although osthole is known for its beneficial biological and pharmacological activities, there is limited information regarding its pesticidal properties [9]. Previous studies have identified its toxicities against the oriental armyworm (*Mythimna separate*), diamondback moth (*Plutella xylostella*), green peach aphid (*M. persicae*), two-spotted spider mite (*T. urticae*), and mosquitoes (*Culex pipiens pallens* and *Aedes aegypti*) [13,36,37,38,39]. In our study, the LC_50_ value against the *T. urticae* decreased from 4.39 to 2.49 mg/mL over a period of 5 days after treatment with osthole, while the LC_50_ value against the *M. persicae* decreased from 4.56 to 2.95 mg/mL. A previous study reported the LC_50_ values of osthole against third-instar *A. aegypti* and *C. p. pallens* larvae as 0.013 mg/mL [39], indicating that osthole potentially possesses stronger pesticidal activity against mosquitoes.

Previous studies have indicated that coumarins have a detrimental effect on the growth, development, and reproduction of arthropods [40,41]. In this study, our findings revealed that osthole treatment had a significant impact on both the developmental time and fecundity of the *M. persicae* and *T. urticae*. Specifically, we observed that osthole treatment prolonged the developmental time of both species and also impaired their fecundity. These results are consistent with previous reports indicating that osthole can extend the developmental time of pests [36].

Prior research has suggested that coumarins act as potent deterrents against arthropods [42,43,44,45]. However, it remains unclear whether osthole also acts as a deterrent. In this study, we present the first evidence that osthole exhibits antifeedant effects against the *T. urticae* and *M. persicae*. It is important to note that the antifeedant activity of osthole against the *T. urticae* and *M. persicae* is limited to a duration of up to 24 h, observed at concentrations of 0.3 and 0.003 mg/mL, respectively. Interestingly, concentrations higher than 0.003 mg/mL do not elicit any antifeedant effects against the *M. persicae*, which is a surprising finding.

Another intriguing discovery in this study is the unexpected observation of osthole’s attraction to oviposition by the *B. dorsalis*. To the best of our knowledge, no published report has described coumarins with oviposition attraction activity. The *B. dorsalis* is a highly polyphagous fruit fly species that infests around 450 plant species worldwide, including *Citrus* spp., which contain osthole [12,17]. Thus, the oviposition attraction activity of osthole towards the *B. dorsalis* and its lack of toxicity might be attributed to the insect’s strong detoxification capacity, developed through a long co-evolutionary process with *Citrus* plants. The preference of the *B. dorsalis* for osthole suggests its potential as a candidate for further exploration as an attractant in the control of the *B. dorsalis*.

## 5. Conclusions

In conclusion, our research has shown that osthole is the predominant coumarin present in CMC. While it does display some pesticidal activity against the *T. urticae* and *M. persicae*, further investigation is needed to enhance its efficacy. Our findings also indicate that osthole acts as a short-term feeding deterrent for female *T. urticae* adults, but potential strategies to prolong its effectiveness should be explored. Furthermore, osthole serves as a powerful attractant for female *B. dorsalis* adults, suggesting its potential application in trapping and monitoring *B. dorsalis* populations. Future research should address the limitations of osthole’s effectiveness and explore ways to optimize its use as a botanical pesticide.

## Figures and Tables

**Table 1:** Efficacy of osthole against female *T. urticae* adults.

Time (d)	Slope ± SE	Chi-Square	*p*-Value	LC_20_ (95% FL) ^1^	LC_50_ (95% FL) ^2^
1	1.44 ± 0.18	14.56	0.002	1.14 (0.001~2.56)	4.39 (1.65~62.77)
2	1.33 ± 0.19	9.52	0.023	0.87 (0.006~1.99)	3.76 (1.43~17.00)
3	1.41 ± 0.19	7.27	0.064	0.86 (0.054~1.77)	3.39 (1.58~8.12)
4	1.50 ± 0.19	4.24	0.237	0.79 (0.46~1.12)	2.90 (2.27~3.67)
5	1.41 ± 0.19	6.03	0.110	0.63 (0.047~1.33)	2.49 (1.08~4.72)

^1^ Concentration of osthole killing 20% and its 95% fiducial limits in mg toxin per mL solution, *n* = 20 nymphs for each LC_20_. ^2^ Concentration of osthole killing 50% and its 95% fiducial limits in mg toxin per mL solution, *n* = 20 nymphs for each LC_50_.

**Table 2:** Efficacy of osthole against *M. persicae* female adults.

Time (d)	Slope ± SE	Chi-Square	*p*-Value	LC_20_ (95% FL) ^1^	LC_50_ (95% FL) ^2^
1	2.07 ± 0.21	7.85	0.049	1.79 (0.69~1.99)	4.56 (2.99~8.01)
2	2.08 ± 0.20	15.67	0.001	1.46 (0.17~2.64)	3.70 (1.80~8.70)
3	2.33 ± 0.22	25.14	0.000	1.37 (0.002~2.77)	3.15 (0.58~10.20)
4	2.36 ± 0.22	23.19	0.000	1.31 (0.008~2.60)	2.98 (0.67~7.93)
5	2.29 ± 0.22	19.50	0.000	1.27 (0.034~2.45)	2.95 (0.90~6.82)

^1^ Concentration of osthole killing 20% and its 95% fiducial limits in mg toxin per mL solution, *n* = 20 nymphs for each LC_20_. ^2^ Concentration of osthole killing 50% and its 95% fiducial limits in mg toxin per mL solution, *n* = 20 nymphs for each LC_50_.

**Table 3:** Efficacy of osthole against *T. urticae* and *M. persicae* nymphs.

Species	Slope ± SE	Chi-Square	*p*-Value	LC_20_ (95% FL) ^1^	LC_50_ (95% FL) ^2^
*T. urticae*	1.18 ± 0.15	4.12	0.25	0.61 (0.35~0.87)	3.12 (2.48~4.01)
*M. persicae*	0.82 ± 0.14	4.23	0.24	0.21 (0.06~0.41)	2.23 (1.58~3.06)

^1^ Concentration of osthole killing 20% and its 95% fiducial limits in mg toxin per mL solution, *n* = 20 nymphs for each LC_20_. ^2^ Concentration of osthole killing 50% and its 95% fiducial limits in mg toxin per mL solution, *n* = 20 nymphs for each LC_50_.

## Data Availability

The data presented in this study are available on request from the corresponding authors.

## Supplementary Materials

The following supporting information can be downloaded at: https://www.mdpi.com/article/10.3390/insects14090725/s1. Table S1. Contents of the coumarins in CMC.

## References

[1] Carvhalo F.P. (2017). Pesticides, environment, and food safety. Food Energy Secur..

[2] Tudi M., Ruan H.D., Wang L., Lyu J., Sadler R., Connell D., Chu C., Phung D.T. (2021). Agriculture development, pesticide application and its impact on the environment. Int. J. Environ. Res. Public Health.

[3] Isman M.B. (2006). Botanical insecticides, deterrents, and repellents in modern agriculture and an increasingly regulated world. Annu. Rev. Entomol..

[4] Isman M.B. (2020). Botanical insecticides in the twenty-first century—Fulfilling their promise?. Annu. Rev. Entomol..

[5] Li Y.M., Jia M., Li H.Q., Zhang N.D., Wen X., Rahman K., Zhang Q.Y., Qin L.P. (2015). *Cnidium monnieri*: A review of traditional uses, phytochemical and ethnopharmacological properties. Am. J. Chin. Med..

[6] Sun Y., Yang A.W.H., Lenon G.B. (2020). Phytochemistry, Ethnopharmacology, Pharmacokinetics and Toxicology of *Cnidium monnieri* (L.) Cusson. Int. J. Mol. Sci..

[7] Cai J., Basnet P., Wang Z., Komatsu K., Xu L., Tani T. (2000). Coumarins from the fruits of *Cnidium monnieri*. J. Nat. Prod..

[8] Liu J., Zhuang H., Mo L., Li Q. (1999). TLC-MS identification of coumarins from extracts of *Cnidium monnieri* (L.) Cusson. J. Instrum. Anal..

[9] Sun M., Sun M., Zhang J. (2021). Osthole: An overview of its sources, biological activities, and modification development. Med. Chem. Res..

[10] Wu H.X., Wang Y.M., Xu H., Wei M., He Q.L., Li M.N., Sun L.B., Cao M.H. (2017). Osthole, a coumadin analog from *Cnidium monnieri* (L.) Cusson, Ameliorates nucleus pulposus-induced radicular inflammatory pain by inhibiting the activation of extracellular signal-regulated kinase in rats. Pharmacology.

[11] Yan J., Xuan W.D., Bian J. (2012). Research progress of osthole. Chin. Pharm..

[12] Zhang Q.Y., Chen H.C., Qin L.P. (2002). Distribution and pharmacological activity of osthole in plant kingdom. World Phyto.

[13] Yan S., Hu Q., Jiang Q., Chen H., Wei J., Yin M., Du X., Shen J. (2021). Simple osthole/nanocarrier pesticide efficiently controls both pests and diseases fulfilling the need of green production of strawberry. ACS Appl. Mater. Interfaces.

[14] Attia S., Grissa K.L., Lognay G., Bitume E., Hance T., Mailleux A.C. (2013). A review of the major biological approaches to control the worldwide pest *Tetranychus urticae* (Acari: Tetranychidae) with special reference to natural pesticides. J. Pest Sci..

[15] Margaritopoulos J.T., Tsitsipis J.A., Zintzaras E., Blackman R.L. (2000). Host-correlated morphological variation of *Myzus persicae* (Hemiptera: Aphididae) populations in Greece. Bull. Entomol. Res..

[16] Paini D.R., Sheppard A.W., Cook D.C., Thomas M.B. (2016). Global threat to agriculture from invasive species. Proc. Natl. Acad. Sci. USA.

[17] Clarke A.R., Armstrong K.F., Carmichael A.E., Milne J.R., Raghu S., Roderick G.K., Yeates D.K. (2005). Invasive phytophagous pests arising through a recent tropical evolutionary radiation: The *Bactrocera dorsalis* complex of fruit flies. Annu. Rev. Entomol..

[18] Helle W., Sabelis M.W. (1985). Spider Mites: Their Biology, Natural Enemies and Control.

[19] van Emden H.F., Harrington R. (2017). Aphids as Crop Pests.

[20] Bass C., Puinean A.M., Zimmer C.T., Denholm I., Field L.M., Foster S.P., Gutbrod O., Nauen R., Slater R., Williamson M.S. (2014). The evolution of insecticide resistance in the peach potato aphid, *Myzus persicae*. Insect Biochem. Mol. Biol..

[21] Jin T., Zeng L., Lin Y., Lu Y., Liang G. (2011). Insecticide resistance of the oriental fruit fly, *Bactrocera dorsalis* (Hendel) (Diptera: Tephritidae), in mainland China. Pest Manag. Sci..

[22] Khajehali J., Van Nieuwenhuyse P., Demaeght P., Tirry L., Van Leeuwen T. (2011). Acaricide resistance and resistance mechanisms in *Tetranychus urticae* populations from rose greenhouses in the Netherlands. Pest Manag. Sci..

[23] Singh K.S., Cordeiro E.M.G., Troczka B.J., Pym A., Mackisack J., Mathers T.C., Duarte A., Legeai F., Robin S., Bielza P. (2021). Global patterns in genomic diversity underpinning the evolution of insecticide resistance in the aphid crop pest *Myzus persicae*. Commun. Biol..

[24] Van Leeuwen T., Vontas J., Tsagkarakou A., Dermauw W., Tirry L. (2010). Acaricide resistance mechanisms in the two-spotted spider mite *Tetranychus urticae* and other important Acari: A review. Insect Biochem. Mol. Biol..

[25] Wei D.D., He W., Lang N., Miao Z.Q., Xiao L.F., Dou W., Wang J.J. (2019). Recent research status of *Bactrocera dorsalis*: Insights from resistance mechanisms and population structure. Arch. Insect Biochem. Physiol..

[26] Zhou H., Wan F., Guo F., Liu J., Ding W. (2022). High value-added application of a renewable bioresource as acaricide: Investigation the mechanism of action of scoparone against *Tetranychus cinnabarinus*. J. Adv. Res..

[27] Bourgaud F., Poutaraud A., Guckert A. (1994). Extraction of coumarins from plant material (*Leguminosae*). Phytochem. Anal..

[28] Mostafiz M.M., Shim J.K., Hwang H.S., Bunch H., Lee K.Y. (2020). Acaricidal effects of methyl benzoate against *Tetranychus urticae* Koch (Acari: Tetranychidae) on common crop plants. Pest Manag. Sci..

[29] Zhang R.M., Jang E.B., He S., Chen J. (2015). Lethal and sublethal effects of cyantraniliprole on *Bactrocera dorsalis* (Hendel) (Diptera: Tephritidae). Pest Manag. Sci..

[30] Li J., Liu J., Chi B., Chen P., Liu Y. (2022). 20E and MAPK signal pathway involved in the effect of reproduction caused by cyantraniliprole in *Bactrocera dorsalis* Hendel (Diptera: Tephritidae). Pest Manag. Sci..

[31] Li H., Ren L., Xie M., Gao Y., He M., Hassan B., Lu Y., Cheng D. (2020). Egg-surface bacteria are indirectly associated with oviposition aversion in *Bactrocera dorsalis*. Curr. Biol..

[32] Capinera J.L. (2001). Handbook of Vegetable Pests.

[33] Chen D., Wang J., Jiang Y., Zhou T., Fan G., Wu Y. (2009). Separation and determination of coumarins in *Fructus cnidii* extracts by pressurized capillary electrochromatography using a packed column with a monolithic outlet frit. J. Pharm. Biomed. Anal..

[34] Gao F., Hu Y., Ye X., Li J., Chen Z., Fan G. (2013). Optimal extraction and fingerprint analysis of *Cnidii fructus* by accelerated solvent extraction and high performance liquid chromatographic analysis with photodiode array and mass spectrometry detections. Food Chem..

[35] Wang J., Chen D., Chen Z., Fan G., Wu Y. (2010). Fast separation and determination of coumarins in *Fructus cnidii* extracts by CEC using poly (butyl methacrylate-co-ethylene dimethacrylate-co-[2-(methacryloyloxy) ethyl] trimethylammonium chloride) monolithic columns. J. Sep. Sci..

[36] Li S., Lv M., Sun Z., Hao M., Xu H. (2021). Optimization of osthole in the lactone ring: Structural elucidation, pesticidal activities, and control efficiency of osthole ester derivatives. J. Agric. Food Chem..

[37] Ren Z., Lv M., Li T., Hao M., Li S., Xu H. (2020). Construction of oxime ester derivatives of osthole from *Cnidium monnieri*, and evaluation of their agricultural activities and control efficiency. Pest Manag. Sci..

[38] Ren Z., Lv M., Sun Z., Li T., Zhang S., Xu H. (2021). Regioselective hemisynthesis and insecticidal activity of C8-hydrazones/ acylhydrazones/sulfonylhydrazones coumarin-type derivatives of osthole. Bioorg. Med. Chem. Lett..

[39] Wang Z., Kim J.R., Wang M., Shud S., Ahn Y.J. (2012). Larvicidal activity of *Cnidium monnieri* fruit coumarins and structurally related compounds against insecticide-susceptible and insecticide-resistant *Culex pipiens pallens* and *Aedes aegypti*. Pest Manag. Sci..

[40] Berenbaum M. (1983). Coumarins and caterpillars: A case for coevolution. Evolution.

[41] Pavela R., Vrchotová N. (2013). Insecticidal effect of furanocoumarins from fruits of *Angelica archangelica* L. against larvae *Spodoptera littoralis* Boisd. Ind. Crops Prod..

[42] Poudel S., Kim Y., Kim Y.T., Lee Y. (2015). Gustatory receptors required for sensing umbelliferone in *Drosophila melanogaster*. Insect Biochem. Mol. Biol..

[43] Poudel S., Lee Y. (2016). Gustatory receptors required for avoiding the toxic compound coumarin in *Drosophila melanogaster*. Mol. Cells.

[44] Stevenson P.C., Simmonds M.S.J., Yule M.A., Veitch N.C., Kite G.C., Irwin D., Legg M. (2003). Insect antifeedant furanocoumarins from *Tetradium daniellii*. Phytochemistry.

[45] Tabashnik B.E. (1987). Plant secondary compounds as oviposition deterrents for cabbage butterfly, *Pieris rapae* (Lepidoptera: Pieridae). J. Chem. Ecol..

