# Supplementary material for: Contact Toxicity, Antifeedant Activity, and Oviposition Preference of Osthole against Agricultural Pests
Source: Insects. 2023 Aug 24;14(9):725. doi: 10.3390/insects14090725 (PMC10531909; doi:10.3390/insects14090725)
Supplement: Supplementary file 1 [file insects-14-00725-s001.zip › insects-2515941-supplementary.pdf]

Table S1 Contents of the coumarins in CMC.

Name	Class	Contents (mg/g)
Osthole	Simple coumarin	11.04 ± 0.87
Imperatorin	Linear furanocoumarin	3.41 ± 0.19
Xanthotoxol	Linear furanocoumarin	1.30 ± 0.085
Isopimpinellin	Linear furanocoumarin	0.64 ± 0.055
5-Methoxypsoralen	Linear furanocoumarin	0.45 ± 0.030
Methoxsalen	Linear furanocoumarin	0.40 ± 0.027
Meranzin (hydrate)	Simple coumarin	0.31 ± 0.025
Auraptenol	Simple coumarin	0.15 ± 0.0098
Columbianadin	Angular furanocoumarins	0.12 ± 0.0075
Angelicin	Angular furanocoumarin	0.060 ± 0.0044
Isogosferol	Linear furanocoumarin	0.011 ±0.00053
